# Ethnopharmacological Properties and Medicinal Uses of *Litsea cubeba*

**DOI:** 10.3390/plants8060150

**Published:** 2019-06-01

**Authors:** Madhu Kamle, Dipendra K. Mahato, Kyung Eun Lee, Vivek K. Bajpai, Padam Raj Gajurel, Kang Sang Gu, Pradeep Kumar

**Affiliations:** 1 Department of Forestry, North Eastern Regional Institute of Science and Technology, Nirjuli 791109, India; madhu.kamle18@gmail.com (M.K.); prgajurel@gmail.com (P.R.G.); 2 School of Exercise and Nutrition Sciences, Deakin University, Burwood, VIC 3125, Australia; kumar.dipendra2@gmail.com; 3 Molecular Genetics Lab, Department of Biotechnology, Yeungnam University, Gyeongsan, Gyeongbuk 38541, Korea; keun126@ynu.ac.kr; 4 Department of Energy and Material Engineering, Dongguk University-Seoul, Seoul 04620, Korea; vbajpai04@gmail.com; 5 Stemforce, 302 Institute of Industrial Technology, Yeungnam University, Gyeongsan, Gyeongbuk 38541, Korea; kangsg@yu.ac.kr

**Keywords:** human health, antimicrobial, essential oils, bioactive compound, conservation

## Abstract

The genus *Litsea* is predominant in tropical and subtropical regions of India, China, Taiwan, and Japan. The plant possesses medicinal properties and has been traditionally used for curing various gastro-intestinal ailments (e.g., diarrhea, stomachache, indigestion, and gastroenteritis) along with diabetes, edema, cold, arthritis, asthma, and traumatic injury. Besides its medicinal properties, *Litsea* is known for its essential oil, which has protective action against several bacteria, possesses antioxidant and antiparasitic properties, exerts acute and genetic toxicity as well as cytotoxicity, and can even prevent several cancers. Here we summarize the ethnopharmacological properties, essentials oil, medicinal uses, and health benefits of an indigenous plant of northeast India, emphasizing the profound research to uplift the core and immense potential present in the conventional medicine of the country. This review is intended to provide insights into the gaps in our knowledge that need immediate focus on in-situ conservation strategies of *Litsea* due to its non-domesticated and dioecious nature, which may be the most viable approach and intense research for the long-term benefits of society and local peoples.

## 1. Introduction

*Litsea cubeba* Pers., Lauraceae, consists of more than 400 species [1] and is predominant in tropical and subtropical regions of India, Southeast Asia, southern China, Taiwan, and Japan. *Litsea* is evergreen, fast growing, and a rare deciduous tree or shrub that attains a height of about 8 m, growing spontaneously in the eastern Himalayas, Assam, Manipur, and Arunachal Pradesh up to an altitude of 2700 m from sea level [2]. In the Assam state of India, the tree is known as “mejankari”, while it is commonly called “May Chang” or “Chinese pepper” in China. *Litsea* plants are the primary source for traditional medicines but they also serve as a secondary source of food for muga silk worms (*Antheraea assama*) [3]. The muga silk (“mejankari pat”) produced from the *Litsea* plant is very attractive and more expensive than the silk produced from other plants [4]. The silk cocoons fed with *Litsea* produce high value silk, which is creamy, glossy, and five times more expensive than silk produced from a primary source of food plant, i.e., *Machilus bombycina* King [3].

**Figure 1:**
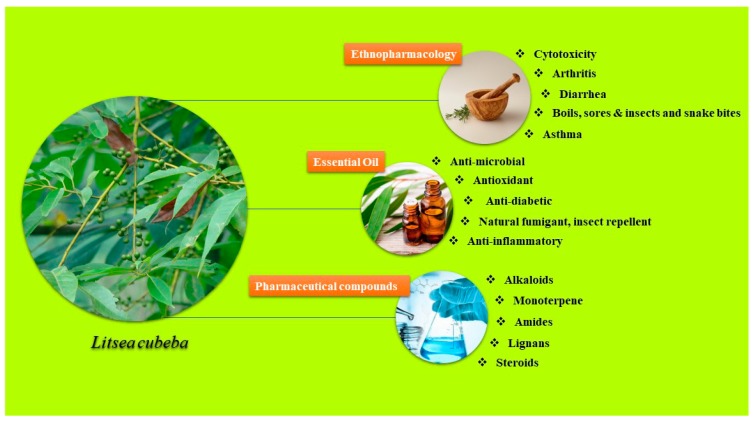
Various ethnopharmacological applications and uses of essential oil of *Litsea cubeba.*

*Litsea cubeba* is a pioneer herb traditionally utilized in medicine. Different extracts from its plant parts, such as bark, leaf, root, and fruits, have been utilized in traditional Chinese medicines for curing various diseases [5,6]. The fresh green fruit is used for culinary purposes like salad preparation, chutneys, pickles, etc. [7]. The *L. cubeba* essential oil (LEO) extracted from fresh fruits contains about 60–90% citral content [6], and is essential oil with volatile compounds having an intense lemon-like, fresh, sweet aroma, and insoluble in water. It was found effective against *Vicia faba,* and weevil (*Bruchus rufimanus*) [8]. China is the one of the largest producers and exporter of *L. cubeba* oil in the world. More than 4.4 million lb of LEO has been produced per year, and three quarters of that production is exported to England, United States, France, Germany, Holland, and other countries [9]. LEO is highly aromatic in nature and extracted from the fresh fruits to exploit as an enhancer of aroma in cosmetic products besides in foods. This is employed as raw material in the production of citral, vitamin A, E, and K, ionine and methylionine, and perfumes, and also to impart antimicrobial and insecticidal properties [10,11,12]. Additionally, LEO is also used as an antifungal agent and bio-insecticides in the storage of grains, foods, archival documents, and/or clothing. The dried fruits are used for several medicinal purposes such as carminative (relieves flatulence), diuretic (aids urine passage), expectorant (aids secretion of sputum), stimulant, stomach ache, antiasthmatic, sedative, antidysentric, and antiseptic [6,9,13]. Some recent studies described the functional properties of *L. cubeba*, such as its therapeutic [9], antimicrobial [14,15], antioxidant [12,16], anti-cancerous [17,18], anti-inflammatory [19], anti-diabetic [20,21,22], and anti-insecticidal activities [23,24,25] (Figure 1). 

## 2. Ethnopharmacological Uses of *Litsea* Species 

Different species of the genus *Litsea* have been used as traditional herbal medicines since 600 A.D. and as sources of important secondary metabolites [26,27,28]. Most *Litsea* species produces odor active compounds while the fruits contain biologically active components that are utilized in various foods as a source of natural ingredients and for flavor [9,29]. The ethnopharmacological properties and uses of *Litsea* species are briefly described in Table 1.

## 3. Essential Oils and Their Applications 

Basically, the essential oils (EOs) are comprised of secondary metabolites intensely present in different parts of the *Litsea cubeba*, such as root, stem, leaf, flower, and fruits. The oils are usually extracted by steam distillation processes. The EOs are chemically a complex mixture of monoterpenes, phenols, and sesquiterpenes [23]. The chemical compounds of EO from different parts of the plant vary in composition, as revealed by different peak area percentage for these compounds in leaves [49], stem bark [50], and flowers [4]. It is important to note that citral rich essential oils are present in fruits while 1,8-cineole predominate the citral content in leaves [51]. The other compounds rich in EO oil from leaves are sabinene and α-pinene [51]. Similarly, *Litsea* EO collected from the different part of the plants from a northeast location of India and analyzed by GC–MS was reported to contain sabinene in maximum proportion in leaf oils (LC1 and LC2) along with other compounds like α-pinene, terpinen-4-ol, α-terpineol, 1,8-cineole, and myrcene. Citronellol and citronellal were dominant in fruit oils (LC3 and LC4) with their respective content of 70% and 10% of the total oil composition. Similarly, geranial (c. 44%) and neral (c. 40%) were also the important components but citronellal was only found to be around 3% in one of the fruit oil samples (LC5) [52].

The chemical composition of the EO of *L. cubeba* has been seen to vary from country to country [14,50]. Despite this, the different EOs possess similar properties and exert antimicrobial, antibacterial, antioxidant, and antiparasitic activity [14,53,54,55]. In addition to this, *Litsea* EO has a peculiar property of insecticidal activity and acts as a repellent against several insects, e.g., cabbage looper (*Trichoplusia ni*), Japanese termite (*Reticulitermes speratus*), mosquito (*Aedes aegypti*), maize weevil (*Sitophilus zeamais*), and red flour beetle (*Tribolium castaneum*), and also possesses nematicidal activity against the pine wood nematode (*Bursaphelenchus xylophilus*) [23,24,56,57]. 

Various agricultural food commodities are attacked by toxigenic fungi across the world [58,59], and pose a serious threat to food safety and security by causing huge crop damages and economic loss. *Aspergillus flavus* is major fungus producing aflatoxins (AFs) that affect several crops and exert carcinogenic, mutagenic, teratogenic, hepatotoxic, and immunosuppressive properties [60,61]. Therefore, the antifungal and antimicrobial effects of *Litsea* EO against several food pathogenic microbes such as *F. verticillioides*, *F. graminearum* and *E. coli* have been investigated by several researchers [15,62,63].

## 4. Pharmaceutical Compounds 

*Litsea cubeba* encompasses a varied number of structurally diverse biologically active compounds, and their uses in traditional medicines and their various functions are listed in Table 2. The major groups of compounds include alkaloids, monoterpenes, sesquiterpenes, diterpenes, flavonoids, amides, lignans, steroids, and fatty acids. These compounds have anticancer, anti-inflammatory, antimicrobial, antioxidant, antidiabetic, and anti-HIV properties, and therefore have immense potential for treating various diseases [64,65]. 

### 4.1. Alkaloids

Around 63 alkaloid compounds have been identified in the genus *Litsea* (few are presented in Table 2). Most of the natural aporphine alkaloids have medicinal properties like antioxidant, antitumor, anticonvulsant, and antiplasmodial properties. These alkaloid compounds and their synthetic derivatives have the potential for curing various diseases [64]. 

### 4.2. Monoterpenes

The maximum proportion of essential oils from *Litsea* species are comprised of monoterpene compounds, i.e., approximately 90% of essential oils. To date, around 20 monoterpene compounds have been extracted from the EO of *L. cubeba* but with varying structures [29,62]. These compounds exhibit a wide range of functions like antioxidant, antifungal, antiasthmatic, and antianaphylactic properties [70]. The monoterpenes can be broadly classified into two categories: menthane and cineole. Menthane has been reported to occur in almost all species of *Litsea* except in *L. coreana* var. *sinensis* (Allen). Further, cineole is present in surplus amounts in *L. mollis* Hemsl. and *L. lancilimaba.*

### 4.3. Sesquiterpenes

Nearly 73 sesquiterpenoid compounds (few are shown in Table 2) have been extracted from different *Litsea* species. These compounds exhibit varying structures, namely aliphatic, monocyclic, bicyclic, and tricyclic sesquiterpenes, along with their oxygenated derivatives. Most of the sesquiterpenes and their derivatives exert natural anti-HIV properties [29].

### 4.4. Diterpenes

This group of compounds is rare in *Litsea* species. Recently, Trisonthi et al. [74] identified and isolated a new cytotoxic diterpene, known as cubelin (235), from the fruits of *L. cubeba* using methanol extract. No other compounds have been reported to exhibit a similar molecular structure as cubelin in *L. cubeba* or any other species of *Litsea*.

### 4.5. Flavonoids 

Flavonoids are another important and major group of compounds present in *Litsea* species. Around 39 compounds have been identified, which include mainly flavones, flavanols, flavanones, flavanonols, anthocyanidins, chalcones, and flavan-3-ols (Table 2); and their glycosidic forms consisting of either glucose, galactose, or rhamnose [62,85]. These compounds are mainly present in *L. coreana, Litsea glutinosa*, and *L. cubeba*. However, some compounds like pinocembrinchalcone (271) and kaempferol 3,4′-di-O-L-rhamnopyranoside (258) were isolated from *L. fruticosa* (Hemsl.) [85]. Flavonoids have therapeutic properties and exhibit beneficial properties like anti-inflammatory, antioxidant, and hepatoprotective activities [11,77,78,79]. 

### 4.6. Amides 

Approximately ten amide compounds have been identified from the genus *Litsea*, as shown in Table 2. The major *Litsea* species from where amides are obtained include *L. acutivena, L. auriculata*, *L. hypophaea, L. greenmaniana*, and *L. cubeba*. The application of amides as chemotaxonomic markers is, however, limited for individual species within the genus *Litsea* [12].

### 4.7. Lignans

Different types of lignans have been reported in *Litsea* species. To date, 35 lignans (few are shown in Table 2) have been extracted from various *Litsea* species: *L. acutivena, L. costalis*, *L. cubeba*, *L. chinpingensis, L. euosma, L. glutinosa, L. greenmaniana, L. grandis, L. gracilipes, L. hypophaea, L. lancifolia, L. lii* var. *nunkao-tahangensis, L. turfosa,* and *L. verticillata* [12].

### 4.8. Steroids 

This group of compounds has limited structural diversity and only about seven steroid compounds have been reported to date from *Litsea* plants.

### 4.9. Fatty Acids

Fatty acids are predominant in *Litsea* species. Some of the major fatty acids present include cinnamic acid, canoic acid, octanoic acid, decenoic acid, dodecenoic acid, myristic acid, stearic acid, oleic acid, and linolenic acid [86]. 

## 5. Functions and Potential Mechanisms of Action

### 5.1. Anticancer Activity 

The EO extracted from the *L. cubeba* fruit has been shown to have cytotoxic effects on human lung, liver, and oral cancer cells [17]. Furthermore, the fumes of oil compounds from *L. cubeba* seeds were detrimental to human NSCLC cells (A549) through the process of cell cycle arrest and apoptosis [18]. Zhang et al. [67] showed the in vitro cytotoxic effects of the alkaloids extracted from *L. cubeba* bark against various human cancer cells, like gastric carcinoma (BGC-823), hepatocellular carcinoma (HepG2), breast cancer (MCF-7), gastric adenocarcinoma (SGC-7901), human skin cancer (SK-MEL-2), and ovarian cancer (SK-OV-3) cells. It has been revealed that the nuclear erythroid-2 related factor (Nrf2) is responsible for controlling the expression of the antioxidant response element (ARE) gene. Therefore, the Nrf2/ARE pathway is supposed to be the potential molecular target to discover chemopreventive medicines [87]. Further, Shen et al. [88] experimented with the selection of EtOH extracts of *L. glutinosa* (ZK-06), *L. monopetala* (ZK-07), and *L. garrettii* (ZK-08), employing a stable ARE luciferase reporter cell line obtained from MDA-MB-231 cells (human breast cancer). It was revealed that the ZK-08 tripled the ARE luciferase activity in comparison to the control, while ZK-06 and ZK-07 showed moderate effects, i.e., two to three times increase in ARE luciferase activity. 

### 5.2. Anti-Inflammatory Activity 

The compound extracted from *Litsea* species has been shown to be effective against gastroenterologia, edema, and rheumatic arthritis, and mainly the species *L. cubeba*, *L. glutinosa, L. akoensis, L. japonica*, and *L. guatemalensis* have been tested for their anti-inflammatory properties [80,89,90]. The inflammatory mediators, nitric oxide (NO) and PGE2, are produced by inducible nitric oxide synthase (iNOS) and cyclooxygenase (COX)-2 enzymes, respectively. The over expression of these two mediators destroys the target tissue during an infection. However, it has been shown that iNOS and COX-2 are not regulated in macrophages, but induce the expression of other pro-inflammatory mediators like IL-6, COX-2, and iNOS for inflammatory response [91]. Therefore, restricting the biosynthesis of prostaglandin and production of NO could potentially treat cancer [92]. The MeOH extract (0.01 mg/mL) was able to prevent the formation of NO and PGE2 in LPS-activated RAW-264.7 macrophages and further declined the release of HOCl and O2^−^ through myeloperoxidase-catalyzed oxidation of chloride [89]. 

### 5.3. Antimicrobial Activity 

Several compounds extracted from *Litsea* species are effective against various pathogenic strains. The EO of *L. cubeba* leaves and fruits from northeast India have shown antimicrobial properties against *S. aureus*, *L. monocytogenes, E. coli*, *P. aeruginosa*, *C. albicans*, and *A. niger*. However, variation in their levels of inhibition was observed, which could be due to variation in the compounds present in the leaves and fruits of *L. cubeba* [52]. Further, antimicrobial activity of the EO from *L. laevigata* was tested in Gram-positive bacteria (*S. aureus, B. subtilis, S. faecalis, S. albus*); Gram-negative bacteria (*E. coli, P. aeruginosa, P. vulgaris,* and *K. aerogenes*), and fungi (*C. albicans* and *A. niger*). The EO was especially effective against Gram-positive bacteria (*S. albus*) and the fungus (*A. niger)* [71].

### 5.4. Antioxidant Activity 

The antioxidant activity of three flavonoids, viz., kaempferol, querctin-3-O-β-D-glucopyranoside, and kaempferol-3-O-β-D-glucopyranoside extracted from *L. coreana* leaves revealed that the kaempferol had the highest activity while kaempferol-3-O-β-D-glucopyranoside showed the least effect [93]. Further, the antioxidant activity from leaf and bark of four different *Litsea* species from India, namely *L. glutinosa*, *L. monopetala, L. assamica*, and *L. laeta* showed that the bark extract of *L. glutinosa* and *L. laeta* had higher metal chelating activity with IC_50_ of 15.25 and 16.14 mg/mL, respectively [94]. Furthermore, the MeOH extracts of the root and stem of *L. elliptica* and *L. resinosa* depicted enhanced antioxidant activity for DPPH (2,2-Diphenyl-1-picrylhydrazyl) radicals with EC_50_ values of 23.99, 41.69, 11.22, and 33.48 mg/L, respectively, against the standard butylated hydroxyl toluene (BHT) [95].

### 5.5. Antidiabetic Activity 

The efficacy of total flavonoids of *Litsea coreana* (TFLC) was investigated for their mechanism to reduce the level of blood glucose in diabetic rats. TFLC was observed to decrease the glucose and lipid levels in the blood and relieved the liver from oxidation stress. In addition, TFLC masked the expression of PTP1B in liver, which resulted in improving the insulin signaling pathway [20]. Similarly, TFLC was further observed to increase the insulin sensitivity, and high-density lipoprotein cholesterol (HDL-C) and superoxide dismutase (SOD) activities. On the other hand, bodyweight, serum free fatty acid, total cholesterol, triglyceride, and low-density lipoprotein cholesterol (LDL-C) content were decreased [12]. 

### 5.6. Anti-HIV Activity 

Several compounds extracted from *L. verticillata*, namely litseachromolaevane B (15-epi-eudesm-4(15)-ene-1β,6β-diol(13), litseagermacrane(14), litseaverticillols A–H (16–23), isolitseaneB(24),1,2,3,4-tetrahydro-2,5-dimethyl-8-(1-methylethy)-l,2-naphthalenediol(25), oxyphyllenodiol B (26), verticillatol (15), hydroxydihydrobovolide (28), 3-epilitsenolide D2 (29), 4-hydroxy-2-methylbut-2-enolide (30), litseabutenolide (31), (+)-epiexcelsin(36), and (+)-5ʹ-demethoxyepiexcelsin (37), have potential anti-HIV activity. These compounds showed growth inhibition of HIV in HOG.R5 cells with IC_50_ values ranging from 2.0 to 34.5 μg/mL [73,96,97], thus providing potential leads to discover medicines for HIV.

## 6. Conservation Strategies 

The suitable growth and conservation of *Litsea* plants are constrained at different stages of life. Seedling development is vital to predict the survival of plants and also to influence the forest regeneration process [14]. *Litsea* seeds possess a long dormancy phase and have the potential to form long-lived seed reserves. Therefore, seed propagation is an inefficient method of propagation and needs involvement of biotechnological interventions for this potentially important medicinal plant for long-term genetic conservation. 

The ecosystem studies have shown that the regeneration of forests depends mainly on two factors, namely the seed rain and the soil seed bank [98,99,100,101]. Seed rain is the result of seed production from several plants within the same community as well as addition of seeds from other neighboring communities [102]. Therefore, seed rain has a significant role in generating new plants and determining the structure, dynamics, and regeneration of any forest community [101,103,104]. The soil seed bank, on the other hand, represents the current and past plant community. It prevents the extinction of local species and aids in the regeneration of the forest [105,106]. Furthermore, the soil seed bank functions as a source of colonizing species and accelerates the process of forest succession [107,108]. In addition, the concept of in situ conservation includes the establishment of different types of nature protection areas. These valuable plants can be conserved in the form of ex situ conservation by creating botanical gardens and arboretums. As an alternative, the germplasm resources storehouse could be restored for the long-term preservation of seeds, pollen, and/or asexual propagules [109]. A rapid clonal propagation system for the conservation of various explant sources (shoot tip, node, leaf, and petiole) of *Litsea cubeba* was developed [110]. Furthermore, in-vitro rooting without growth-regulator is possible and over 100 plantlets have been successfully developed in the glasshouse. 

## 7. Conclusions and Future Prospects

The present review has discussed the ethnopharmacological properties of *Litsea cubeba* compounds having the potential to cure various ailments because of inherent anticancer, antimicrobial, anti-inflammatory, antioxidant, antidiabetic, and anti-HIV properties. However, the underlying mechanism and their mode of action are not well researched and established. Further in vitro and in vivo genotoxic experiments of *Litsea* need to be evaluated to entitle its ethnomedical values. The in-depth exploration of *Litsea cubeba* for its various withstanding pharmacological properties can potentially be employed as an initiative to discover new drugs to treat serious diseases like cancer and HIV. Furthermore, it is also considered high time to combine biological research activity with clinical applications to gain insights into the mechanisms of action, drug reactions, and other health related issues associated with the consumption of crude extracts of the plant. Therefore, research involving clinical evaluation along with conservation strategies is imperative for long term benefits to society.

## Figures and Tables

**Table 1:** Ethnopharmacological properties of *Litsea* species and their uses.

Country	Species	Plant Parts	Ethnopharmacological Properties	Solvent/Ratio/Dose Administered	Reference
Taiwan	*L. akoensis* Hayata	Stem bark	Cytotoxicity, antimicrobial activity	15–30 μL of the oil dissolved in dimethylsulfoxide (DMSO) inoculated to plates with test microorganisms	[30]
China, Taiwan, Indochina	*L. acutivena* Hayata	Leaves and twigs	Oil for antimicrobial	50 μL of 1 mg/mL MTT administered against A549 and HT-29 cells	[31]
India, China, Taiwan, Indonesia, and other parts of Southeast Asia	*L. cubeba* (Lour.) Pers.	Fruits	Pain reliever, promotes blood circulation, relieves stomach distension, asthma, demesia, diarrhea, turbid urine, and traumatic injury	-	[28,32,33,34]
		Roots	Relieves cold, stomachache, headache, dermatophytosis, and arthralgia	-	
		Leaves	Promotes blood circulation, cures mammitis, heals hemostasis, sores furuncle, insect and snake bites, cures myocardial infarction in Wistar rats	100 to 200 mg/kg of extract daily for a period of 21 days in rats	
India	*L. chinensis* (Gaertn.) Sonner.	Fruit, leaves, stem	Activates sexual behavior	500 mg/kg of extract to male rats	[35]
Malaysia, Indonesia, Philippines, Taiwan	*L. garciae* S. Vidal	Fruits	Antifungal, antioxidant	Samples (0.1 g) extracted for 2 h with 80% methanol	[36]
Southern Korea, Japan	*L. japonica* (Thunb.) Jussieu	Leaves	Antioxidative, anti-inflammatory	Assay with IC_50_ values of 149 and 58 μM	[37,38]
India (Eastern Himalaya)	*L. laeta* (Nees) Hook. f.	Leaves	Fuelwood	-	[39]
Nepal, India, Bangladesh, Burma, China	*L. monopetala* (Roxb.) Pers	Barks, leaves, roots, trunk	Cures gonorrhea, skin diseases, boil, diarrhea, and dislocation, antimicrobial	Fungal growth inhibition at 150–250 µL/L with fumigation	[40,41,42]
Taiwan	*L. nakaii* Hayata	Leaves	Antimicrobial	15–30 μL of the oil in DMSO applied to microbial plates	[43]
Indonesia	*L. odorifera* Val.	Leaves	Anti HSV-1	-	[44]
India	*L. polyantha* Juss.	Barks and roots	Effective in pains, bruises, fractures, diarrhea	-	[45]
China	*L. rotundifolia* Hemsl.	Roots	Treating rheumatic pain	-	[33]
India, Nepal, Bhutan, Vietnam, Bangladesh, Myanmar, China	*L. salicifolia* (J. Roxb. ex Nees) Hook. f.	-	Fruits for bone fracture, stomach disorder	-	[46,47]
Malaysia (Sarawak)	*L. turfusa* Kosterm.	Ground barks	Antifungal, antitumor	-	[48]

**Table 2:** Compounds isolated from *Litsea cubeba* and their properties.

Compounds	Function	Reference
**Alkaloids**		
(–)-8-O-Methyloblongine; (–)-Litcubine; (–)-Litcubinine; (–)-Magnocurarine; (–)-Oblongine; (+)-Isoboldine β-N-oxide;(+)-8-Methoxyisolaurenine-N-oxide; (+)-N-(Methoxycarbonyl)-N-(norboldine/norglaucine/norlauroscholtzine/norglaucine/norbulbodione/nordicentrin/norisocorydione/norpredicentrine); Actinodaphnine; Isoboldine; Atheroline; Boldine; Cassameridine; Cassythicine; Coclaurine; Corydine; Corytuberine; Dicentrine; Dicentrinone; Glaucine; Glaziovine; Isocorydine; Isodomesticine; Juziphine; Laetanine; Laetine; Lancifoliaine; Laurelliptine; Laurolitsine; Laurotetanine; Lindcarpine; Litebamine; Litsedine; Litseferine; Litseglutine B; Magnoflorine; N,O-Dimethylharnovine; N-Acetyllaurolitsine; N-Allyllaurolitsine; N-Methylcoclaurine; N-Methyllaurotetanine; N-Methyllindcarpine; Norcorydine; Nordicentrine; Norisoboldine; Norisocorydine; Norjuziphine; Oxoushinsunine; Pallidine; Phanostenine; Predicentrine; Reticuline; Sebiferine; Ushinsunine; Xanthoplanine; Butanolides and Butenolactone	Antioxidant, antiplatelet, antitumor, anticonvulsant, and antiplasmodial effects	[12,13,66,67,68,69]
**Monoterpenes**		
Camphene; Bornylacetate; DL-Carvone; 1,8-Cineole; Citronellal; Citronellol; p-Cymene; Geranial; Geranyl acetate; Geraniol; Limonene; Linalool; β-Myrcene; Neral; Nerol; Neryl acetate; (E)-β-Ocimene; (Z)-β-Ocimene; β-Phellandrene; α-Pinene; β-Pinene; α-a-Isopulegol; Sabinene; cis-Sabinene hydrate; α-Terpineol; Terpinen-4-ol; Terpinolene; α-Terpinylacetate; Litseacubebic acid	Antibacterial activity	[29,62,70]
**Sesquiterpenes**		
α-Amorphene; Aphanamol II; Aromadendrene; Bulnesol; α-Cadinene; β-Cadinene; γ-Cadinene; δ-Cadinene; α-Cadinol; β-Caryophyllene; Chromolaevanedione; α-Copaene; Isocurcumol; Elemol; β-Elemene; γ-Elemene; α-Eudesmol; β-Eudesmol; γ-Eudesmol; Germacrene; α-Humulene; Humulene oxide; Indonesiol; Ledene	Defensive roles	[29,71,72,73]
**Diterpenes**		
Cubelin ((þ)-6-(4-hydroxy-4-methyl-2-pentenoyl)-4,6-dimethyl-5-(3-methyl 2-butenyl)-1,3-cyclohexadienecarbaldehyde);trans-Phytol	Antioxidative, antifungal, antiasthmatic, anti-anaphylactic properties	[74,75,76]
**Flavonoids**		
Flavones; flavanols; flavanones; flavanonols; anthocyanidins; chalcones; flavan-3-ols	Anti-inflammatory, antioxidant, and hepatoprotective activities	[11,77,78,79]
**Amides**		
cis-N-Feruloyl-3-methoxytyramine; N-Feruloyl-3-methoxytyramine; 3-Methoxy-N-sinapoyltyramine; N-trans-3,4-methylenecinnamoyl-3-methoxytyramine; Cubebamine A; 1,2-dihydro-6,8-dimethoxy-7-1-(3,5-dimethoxy-4-hydroxyphenyl)-N1,N2-bis-(2-(4-hydroxyphenyl)ethyl)-2,3-naphthalene dicarboxamide; N-cis-3,4-methylenedioxycinnamoyl-3-methoxytyramine	Anticancer effects	[6,68,80,81]
**Lignans**		
Eugenol; syringaresinol; 9,9′-O-di-(E)-feruloyl-(+)-secoisolariciresinol; 9,9′-O-di-(E)-feruloyl-5,5′-(+)-dimethoxysecoisolariciresinol; balanophonin B; (+)-medioresinol; Lancifolin A; cyclolignan; Dehydrodieugenol; Dehydrodiisougenol; Grandisin; (+)-Eudesmin; (+)-Epiexcelsin; Biseugenol A, B; syringaresinol; Glochidioboside	Antioxidant and anticancer effects	[80,82]
**Steroids**		
β-sitostenone; Daucosterol; β-Sitosterol; Sepesteonol, 5,6-Epoxystigmastan-3-ol; Stigmasterol; 6-O-Palmitoyl-β-sitosteryl-D-glucoside		[6,83]
**Fatty acids**		
Capric acid; cis-Dec-4-enoic acid; cis-Dodec-4-enoic acid (Linderic acid); cis-Tetradec-4-enoic acid (Tsuzuic acid); Hexadecenoic acid; Lignoceric acid; Lauric acid; Linoleic acid; Myristic acid; Oleic acid; Palmitic acid; Ethyl palmitate; Stearic acid; Ethyl stearate; Litseacubebic acid; 2,6-Dimethyl-6-hydroxy-2E,4E-hepta-2,4-dienal; 6,7-Dihydroxy-3,7-dimethyl-oct-2-enoic acid	Antidiabetic effects	[62,81,84]
